# Reclassification of the genus Dysgonomonas and description of Dysgonomonas reticulitermitis sp. nov. and Viscerimonas tarda gen. nov., sp. nov. from the gut of the subterranean termite Reticulitermes speratus

**DOI:** 10.1099/ijsem.0.007031

**Published:** 2026-01-19

**Authors:** Kazuki Takahashi, Atsushi Hisatomi, Kosuke Mori, Masahiro Yuki, Satoko Noda, Yuichi Hongoh, Moriya Ohkuma, Mitsuo Sakamoto

**Affiliations:** 1 School of Life Science and Technology, Institute of Science Tokyo, Meguro ku, Tokyo 152-8550, Japan; 2 Microbe Division/Japan Collection of Microorganisms, RIKEN BioResource Research Center, Tsukuba 305-0074, Japan; 3 Graduate School of Life and Environmental Sciences, University of Yamanashi, Yamanashi 400-8510, Japan; 4 Graduate School of Science and Engineering, Ibaraki University, Mito 310-8512, Japan; 5 NODAI Culture Collection Center, Tokyo NODAI Research Institute, Tokyo University of Agriculture, Setagaya-ku, Tokyo 156-8502, Japan

**Keywords:** *Dysgonomonas*, phylogenomic analysis, termite

## Abstract

The genus *Dysgonomonas*, established in 2000 and currently comprising nine isolated species, belongs to the order *Bacteroidales*. Despite its increasing ecological and clinical relevance, the genus remains taxonomically ambiguous, and criteria for genus-level classification have not been systematically assessed. In this study, we re-evaluated the taxonomy of *Dysgonomonas* using core genome phylogeny, average amino acid identity and percentage of conserved proteins. These genome-based metrics and physiological data revealed that the genus *Dysgonomonas* comprises at least three distinct genus-level lineages, and (i) *Dysgonomonas sensu stricto* represented by *Dysgonomonas gadei*, (ii) *Indolivaga* gen. nov. represented by *Dysgonomonas capnocytophagoides* and (iii) *Pseudodysgonomonas* gen. nov. represented by *Dysgonomonas massiliensis* are proposed. In addition, two strains MK137_Hg11^T^ and MK137_Hg34^T^, facultative anaerobic bacteria, were isolated from the gut of the subterranean termite *Reticulitermes speratus*. On the basis of the collected data, we propose that strain MK137_Hg11^T^ represents a novel species within *Dysgonomonas*, while strain MK137_Hg34^T^ represents a novel species belonging to a fourth, previously unrecognized genus within the family *Dysgonomonadaceae*. We therefore propose the names *Dysgonomonas reticulitermitis* sp. nov. (type strain MK137_Hg11^T^=JCM 35194^T^=DSM 118089^T^) and *Viscerimonas tarda* gen. nov., sp. nov. (type strain MK137_Hg34^T^=JCM 35195^T^=DSM 118090^T^).

## Introduction

The genus *Dysgonomonas* (family *Dysgonomonadaceae*, order *Bacteroidales*) comprises facultatively anaerobic, fermentative bacteria that have been isolated from diverse environments, including human clinical samples, sea sand, microbial fuel cells, human gut and termite hindguts [1,8]. Members of this genus are known for producing short-chain fatty acids such as acetate, lactate and propionate. Several species also possess a wide array of carbohydrate-active enzymes (CAZymes), suggesting the potential roles in polysaccharide degradation.

Clinically, *Dysgonomonas* species have been recognized as opportunistic pathogens, particularly in immunocompromised individuals. In parallel, the increased use of high-throughput sequencing has revealed *Dysgonomonas*-related sequences in a variety of terrestrial environments [9]. Among these, insect guts – particularly those of termites and cockroaches – have emerged as one of the most prominent sources of these sequences [10,19]. Termites feed exclusively on dead plant matter and play a crucial role in the terrestrial carbon cycle, particularly in temperate to tropical regions [20,21]. Recently, four isolates from the gut of the subterranean termite *Reticulitermes flavipes* have been reported, some of which have a high abundance of genes for oligo- and polysaccharide utilization [9,22, 23]. Similarly, five isolates from the American cockroach *Periplaneta americana* have been shown to encode diverse CAZyme repertoires [24].

Despite this increasing interest, only two species from insect guts have been validly described: *Dysgonomonas macrotermitis*, from the fungus-growing termite *Macrotermes barneyi* [7], and *Dysgonomonas termitidis*, from *Reticulitermes speratus* [6]. Furthermore, the current taxonomic framework of the genus *Dysgonomonas* remains poorly resolved, and its classification criteria at the genus level have yet to be systematically examined. In this study, based on genome-based metrics and phylogenomic analyses, we propose a reclassification of the genus *Dysgonomonas* into three distinct genera: the first containing the type species of this genus, *Dysgonomonas gadei*; the second including *Dysgonomonas capnocytophagoides*, for which *Indolivaga* gen. nov. is proposed; and the third including ‘*Dysgonomonas massiliensis*’, for which *Pseudodysgonomonas* gen. nov. is proposed. In addition, we characterize two strains, MK137_Hg11^T^ and MK137_Hg34^T^, isolated from *R. speratus*. Based on phylogenetic and genomic evidence, we propose that MK137_Hg11^T^ represents a novel species within the genus *Dysgonomonas*, while MK137_Hg34^T^ represents a novel species belonging to a fourth, previously unrecognized genus within the family *Dysgonomonadaceae*.

## Isolation and ecology

The hindgut fluid of *R. speratus* was homogenized in Trager’s Solution U [25] and plated onto Trypticase Soy Broth (Sigma-Aldrich) solidified with gellan gum and supplemented with the vitamin solution from JCM medium no. 197. After 30 days of incubation at 30 °C under microoxic conditions using the AnaeroPack-MicroAero system (Mitsubishi Gas Chemical), a number of strains were isolated, and among them, strains MK137_Hg11^T^ and MK137_Hg34^T^, which likely represented novel species within the genus *Dysgonomonas*, were selected by the 16S rRNA gene sequence analysis [26].

## 16S rRNA gene phylogeny

**Fig. 1.**
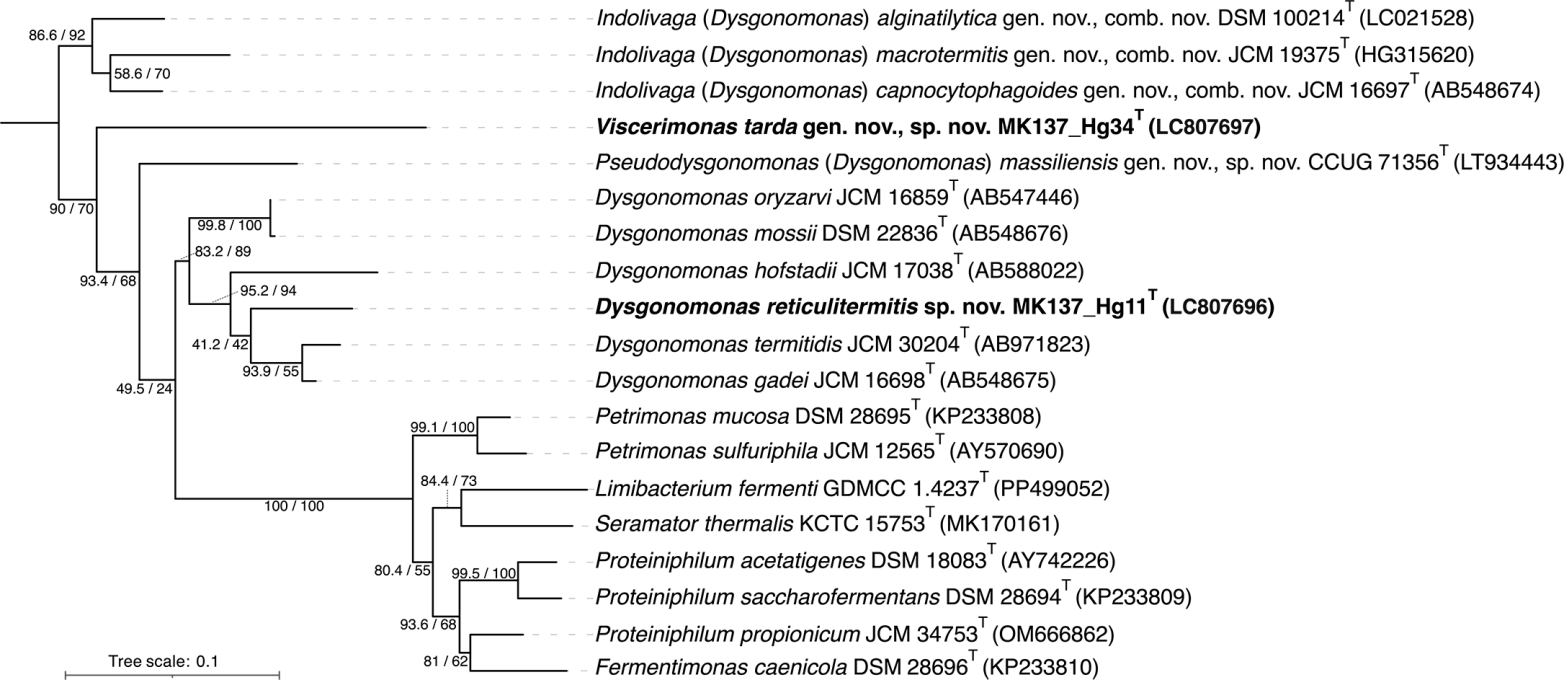
Maximum-likelihood phylogenetic tree of 16 validly published *Dysgonomonadaceae* species based on the 16S rRNA gene sequences. The tree was rooted with *Parabacteroides faecis* (AB739697) and *Parabacteroides hominis* (MT905166) as the outgroup. SH-aLRT value (left) and ultrafast bootstrap support value (right) are shown at branch nodes.

The 16S rRNA genes were amplified directly from the colonies by PCR using Ex Taq DNA polymerase (TakaraBio) and primer sets 27F (5′-AGAGTTTGATCCTGGCTCAG-3′) and 1390R (5′-AGGCCCGGAACGTATTCAC-3′). The similarity of each sequence was analysed by blastn against the EzBioCloud databases [27]. Strain MK137_Hg11^T^ showed the highest 16S rRNA gene sequence similarity to *D. gadei* (95.0%). Strain MK137_Hg34^T^ showed the highest 16S rRNA gene sequence similarity to *D. capnocytophagoides* (90.0%), a value below the commonly accepted genus-level threshold (92–95%). The pairwise sequence similarity between strains MK137_Hg11^T^ and MK137_Hg34^T^, calculated using clustal Omega v1.2.4 [28], was 89.8%. The curated 16S rRNA gene sequence alignment from the All-Species Living Tree Project database LTP_10_2024 [29] was used as a reference framework for phylogenetic analysis. Into this alignment, the 16S rRNA gene sequences of strains MK137_Hg11^T^, MK137_Hg34^T^ and other relevant taxa (e.g. ‘*D. massiliensis*’) were incorporated using ARB [30]. After trimming the alignment with the ‘gappyout’ option in TrimAL v1.4rev22 [31], a final dataset of 1,523 aligned positions was used for tree reconstruction. A maximum-likelihood tree was constructed using IQ-TREE v 2.2.2.7 with the TPM3+I+R3 model and the ultrafast bootstrap and SH-aLRT tests (1,000 replicates) [32]. The phylogenetic analysis based on 16S rRNA gene sequences placed strain MK137_Hg11^T^ within a clade that included *D. gadei*, *D. termitidis* and *Dysgonomonas hofstadii* (Fig. 1). The node defining this group received a SH-aLRT value of 95.2% and an ultrafast bootstrap value of 94%. In contrast, strain MK137_Hg34^T^ formed a distinct lineage on a long branch, and its phylogenetic position relative to other genera remained unresolved, as indicated by the low support values on deep nodes within the tree. This topology suggests that strain MK137_Hg11^T^ is a member of the *Dysgonomonas* clade, while strain MK137_Hg34^T^ may belong to a separate, deeper-branching lineage.

## Genome features

The whole genomes of strains MK137_Hg11^T^ and MK137_Hg34^T^ were sequenced using the Illumina MiSeq platform. The Illumina MiSeq 2×300 bp paired-end sequencing library was prepared using the QIAseq FX DNA Library Kit. MiSeq reads were trimmed and filtered using fastp v0.23.4 [33]. Quality trimmed reads were assembled to contigs using SPAdes v3.14.1 [34], and gene prediction and annotation were performed using NCBI Prokaryotic Genome Annotation Pipeline (PGAP). Genome completeness and contamination were assessed using CheckM2 v1.0.2 [35]. The strains MK137_Hg11^T^ and MK137_Hg34^T^ had completeness levels of 99.9 and 100%, with contamination levels of 0.1 and 0.6%, respectively. The genome of strain MK137_Hg11^T^ was 3,821,473 bp with a G+C content of 39.5 mol% and contained 3,074 protein-coding sequences (CDSs). The strain MK137_Hg34^T^ chromosome was 3,796,581 bp with a G+C content of 43.3 mol% and contained 3,170 CDSs. Digital DNA–DNA hybridization (dDDH) values were estimated using the genome-to-genome distance calculator v3.0 with blast+ as the local alignment tool [36], and average nucleotide identity (ANI) values were calculated using the OrthoANI [37]. The dDDH and ANI results among strains MK137_Hg11^T^, MK137_Hg34^T^ and related species are summarized in Table S1, available in the online Supplementary Material. The dDDH and ANI values among strains MK137_Hg11^T^, MK137_Hg34^T^ and related species were below the species demarcation thresholds (30% for dDDH and 79% for ANI), supporting their classification as novel species.

**Fig. 2.**
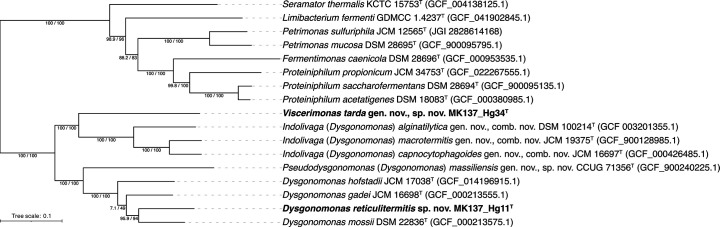
Maximum-likelihood phylogenomic tree of isolates belonging to the family *Dysgonomonadaceae* based on concatenated amino acid sequences of single-copy marker genes. The tree was rooted with *Coprobacter fastidiosus* (GCF_000473955.1) and *Coprobacter secundus* subsp. *similis* (GCF_015097275.1) as the outgroup. SH-aLRT value (left) and ultrafast bootstrap support value (right) are shown at branch nodes.

For phylogenomic tree reconstruction, 74 single-copy bacterial marker proteins were aligned and concatenated using GToTree v1.8.2 [38]. A maximum-likelihood tree was then constructed in IQ-TREE v2.2.5 using the LG+F+I+R4 model selected by its model finder and evaluated using ultrafast bootstrap and SH-aLRT tests with 1,000 replicates. The strains MK137_Hg11^T^ and MK137_Hg34^T^ formed a cluster with other *Dysgonomonas* species (Fig. 2). However, these strains were separated into two clades within the genus *Dysgonomonas*. Strain MK137_Hg11^T^ was closely related to *D. gadei*, *D. hofstadii* and *Dysgonomonas mossii*. Strain MK137_Hg34^T^ was a sister group to *D. capnocytophagoides*, *D. macrotermitis* and *Dysgonomonas alginatilytica*.

**Fig. 3.**
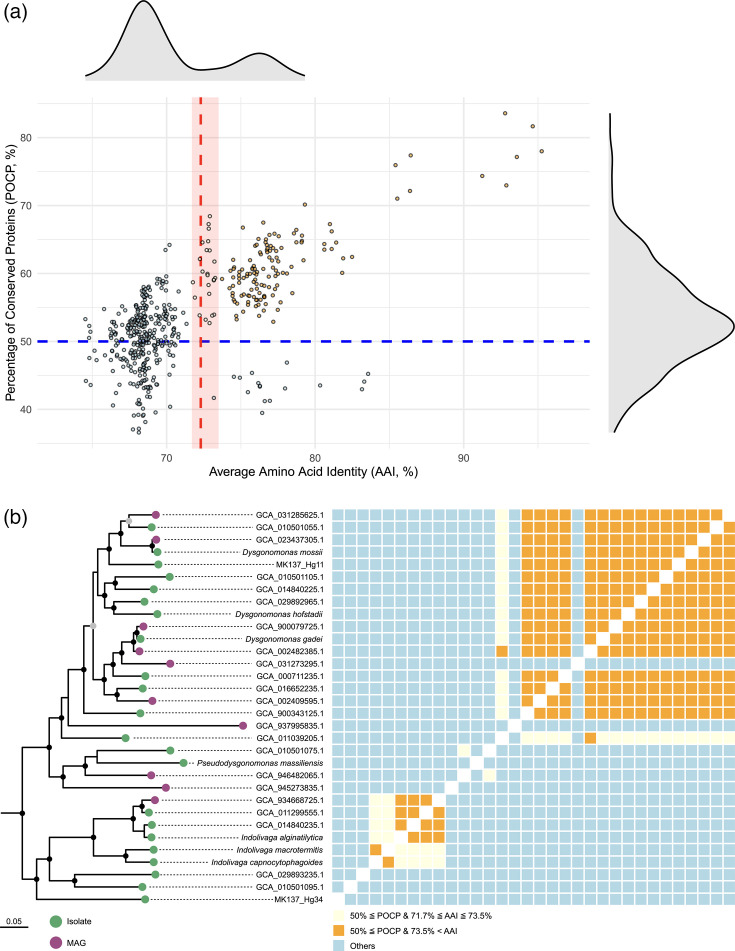
(**a**) Relationship between AAI and POCP values based on pairwise whole-genome comparisons. Each point represents a comparison between two genomes. The red dashed line indicates the local minimum of the AAI distribution estimated with kernel density estimation using an adjustment parameter (*adjust*) of 1. The light red band represents the range of local minima obtained by varying the *adjust* value in the kernel density estimation. (**b**) Maximum-likelihood phylogenomic tree of isolates and MAGs assigned to the genus *Dysgonomonas*, constructed from a concatenated alignment of single-copy marker gene amino acid sequences. The tree was rooted with *Coprobacter fastidiosus* (GCF_000473955.1) and *Coprobacter secundus* subsp. *similis* (GCF_015097275.1) as the outgroup. Nodes with SH-aLRT support values ≥80% and ultrafast bootstrap support values ≥95% are indicated by solid black circles; nodes with either SH-aLRT≥80% or ultrafast bootstrap≥95% are indicated by solid grey circles. Outer tiles correspond to the AAI–POCP relationship shown in (**a**), with colours indicating the combined criteria.

Average amino acid identity (AAI) values and percentage of conserved proteins (POCP) were also calculated using EzAAI v1.2.3 [39] and POCP-nf [40] with default settings, respectively. The AAI and POCP values among strains MK137_Hg11^T^, MK137_Hg34^T^ and related species are summarized in Table S2. The POCP values between MK137_Hg34^T^ and related species were below the genus-level threshold of 50%, suggesting that strain MK137_Hg34^T^ may represent a member of a distinct genus. To clarify genus-level boundaries within this group, we examined whether AAI and POCP values exhibited bimodal distributions (Fig. 3a) in comparisons with genomes within the genus *Dysgonomonas*, including metagenome-assembled genomes (MAGs). Although POCP values did not show a clear bimodal pattern, AAI values did, with a local minimum estimated at ~72%, identified within the 60–80% range using kernel density estimation with the density function in R v4.4.2 and default parameters. To assess the robustness of the estimated local minimum, we varied the bandwidth adjustment parameter (adjust) from 0.5 to 2.0. The resulting local minima consistently fell within the 71.7–73.5% range. When AAI was recalculated using amino acid identities of 128 conserved single-copy genes, the bimodal pattern became more pronounced (Fig. S1). Applying the commonly used 50% threshold for POCP, genome pairs that met both the AAI and POCP criteria were broadly grouped into two major monophyletic clades in the phylogenetic tree – corresponding to the *D. gadei* group and the *D. capnocytophagoides* group – excluding some MAGs (Fig. 3b). These results suggest that the genus *Dysgonomonas* may be subdivided into multiple genera based on genome-wide signatures.

## Physiology and chemotaxonomy

MK137_Hg11^T^ was grown on Brucella blood agar with haemin and menadione (JCM medium no. 677) and MK137_Hg34^T^ was on EG agar supplemented with 5% (v/v) horse blood for 3–7 days at 30 °C under microoxic conditions using the AnaeroPack-MicroAero system or a H_2_/CO_2_/N_2_ (1:1:8, by vol.) gas mixture. *D. macrotermitis* JCM 19375^T^ was used as a reference strain. This strain was obtained from the Japan Collection of Microorganisms (JCM), RIKEN BioResource Research Center, Tsukuba, Japan. Cells cultivated for 2 days at 30 °C under microoxic conditions in an appropriate medium were subjected to Gram staining. Cell morphology was examined using phase-contrast microscopy (Biophot, Nikon). Cells of strain MK137_Hg11^T^ on Brucella blood agar were 1.5–5.0×0.5 µm in size and occurred as single, Gram-stain-negative, bacilliform or wavy rods. The morphology of strain MK137_Hg34^T^ on EG agar was 2.0–5.0×0.8 µm in size and occurred singly or in short chains, Gram-stain-negative, bacilliform or wavy rods. The strains were incubated at different temperatures (8, 15, 25, 30, 37, 42 and 45 °C) to examine bacterial growth. All strains except *D. macrotermitis* JCM 19375^T^ grew at 25–30 °C with optimum growth at 30 °C, whereas *D. macrotermitis* grew at 25–37 °C with an optimum at 37 °C. Therefore, subsequent tests were performed at the optimal growth temperature for each strain. The isolates were cultivated to determine the optimum pH (at pH 3.0, 4.0, 5.0, 6.0, 6.5, 7.0, 7.5 and 8.0). Strains MK137_Hg11^T^ and *D. macrotermitis* JCM 19375^T^ grew at pH 6.0–8.0 (optimum, pH 7.0–7.5) and strain MK137_Hg34^T^ grew at pH 6.5–8.0 (optimum, pH 6.5–7.5). All isolates were non-motile, as confirmed by a motility test in which tubes of semisolid nutrient agar medium (0.15% or 0.5% agar) were stab-inoculated and incubated for 7 days [41]. Bile resistance was assessed by culturing bacteria in peptone–yeast extract–glucose (PYG) medium with 0.5% or 2% (w/v) Difco-Oxgall (BD). Strains MK137_Hg11^T^ and MK137_Hg34^T^ did not grow in the presence of 0.5% Oxgall, whereas *D. macrotermitis* JCM 19375^T^ was able to grow under the same condition.

Catalase activity was assessed by observing gas formation after adding fresh cells to a 3% H₂O₂ solution, with only strain MK137_Hg11^T^ testing positive. Biochemical reactions were evaluated in triplicate using the API ZYM system and Rapid ID 32A anaerobe identification kit (bioMérieux), following the manufacturer’s instructions. The ability to utilize various carbon sources, including d-glucose, cellobiose, maltose, l-arabinose, d-xylose, l-rhamnose, sucrose, lactose, trehalose, d-mannitol and CM-cellulose, was evaluated in yeast casitone fatty acids (YCFA) medium supplemented with 0.5% (w/v) of each substrate for 7 days by measuring turbidity. The results are summarized in Table 1 and the species description.

**Table 1.** Differential characteristics of strains MK137_Hg11^T^, MK137_Hg34^T^ and related species of the genus *Dysgonomonas* Strains: 1, MK137_Hg11^T^; 2, *D. mossii* CDC F9489^T^ (unless indicated otherwise, data were taken from [3]); 3, *D. hofstadii* MX 1040^T^ [5]; 4, *D. gadei* CCUG 42882^T^ [1]; 5, *D. oryzarvi* Dy73^T^ [4]; 6, *D. termitidis* N-10^T^ [6]; 7, ‘*D. massiliensis*’ Marseille-P4356^T^ [8]; 8, *D. alginatilytica* HUA-2^T^ [2]; 9, *D. macrotermitis* JCM19375^T^; 10, *D. capnocytophagoides* CCUG 17996^T^ [1]; 11, MK137_Hg34^T^. +, Positive; −, negative; +/−, may or may not be produced; w, weak; v, variable; nd, no data available; A, acetate; F, formate; L, lactate; P, propionate; S, succinate.

	1	2	3	4	5	6	7	8	9	10	11
Cell size (µm)	1.5–5.0×0.5	nd	nd	nd	1.0×1.5	1.6×8.3	0.6×0.6	0.5–0.6×1.5–3.0	0.5–1.0×2.0–20	nd	2.0–5.0×0.8
DNA G+C content (mol%)	39.5	38.5	39.5^*b*^	39.5^*c*^	37.5	41.8	37.3	37.5	38.5^*d*^	37.5^*e*^	43.3
Genome size (Mb)	3.82	3.95^*a*^	5.04^*b*^	5.18^*c*^	nd	nd	3.47	5.12	4.66^*d*^	4.38^*e*^	3.8
Cell morphology	Bacilliform or wavy rods	Coccobacilli to short rods	Coccobacilli to short rods	Coccobacilli	Coccoid- to short-rod-shaped	Rod	Cocci	Rod	Bacilliform or wavy rods	Coccobacilli to short rods	Bacilliform or wavy rods
**Growth at/with:**											
25 ℃	+	+	+	+	nd	+	+	+	+	nd	+
37 ℃	−	+	+	+	+	+	+	+	+	nd	−
pH 6.0	w	nd	nd	nd	nd	nd	+	+	w	nd	−
pH 8.0	+	nd	nd	nd	nd	nd	+	+	+	nd	w
**Resistance to bile**	−	+	nd	+	−	−	−	−	+	+	−
**Growth on:**											
d-Glucose	+	+	+	+	+	+	+	+	+	+	+
Cellobiose	+	+	+	+	+	−	nd	nd	+	nd	−
Maltose	+	+	+	nd	+	+	+	+	+	+	−
l-Arabinose	−	+	−	+	+	w	+	+	+	+	−
Lactose	+	+	+	+	+	+	+	+	+	+	+
d-Mannitol	−	+	−	−	−	−	+	−	−	−	−
l-Rhamnose	−	+	−	+	+	w	−	+	+	nd	+ (v)
Sucrose	+	+	+	+	+	+	−	+	−	+	−
Trehalose	+	+	+	+	−	−	−	−	−	−	−
d-Xylose	+	+	+	+	+	w	+	+	+	+	+
CM-cellulose	−	nd	nd	nd	nd	w	nd	nd	−	nd	−
**Enzyme activity:**											
*α*-Arabinosidase	−	+/−	+	+	+	+	nd	nd	+	+	+
*α*-Fucosidase	+	+	+	+	+/−	+	−	+	−	−	−
*α*-Galactosidase	+	+	+	+	+	+	+	+	+	+	−
*β*-Galactosidase	+	+	+	w	+	+	−	−	+	+	+
*β*-Glucosidase	+	+	+	+	+	+	+	+	+	+	−
*β*-Glucuronidase	+/−	−	−	+/−	−	−	−	−	+/−	−	−
*β*-Galactosidase 6 phosphate	−	+	nd	−	+	+	nd	nd	+	+	−
*N*-Acetyl-*β*-glucosaminidase	+	+	+	+	+	+	+	+	+	−	−
Leucine arylamidase	+/−	−	−	−	−	−	−	−	−	−	−
Trypsin	+	nd	nd	+	nd	nd	−	−	−	−	−
*α*-Chymotrypsin	−	nd	nd	+	nd	nd	+	−	+	−	−
Alkaline phosphatase	+	+	+	+	+	+	−	+	+	+	+
Indole production	+	+	+	+	+/−	+	nd	−	−	+/−	−
End products from glucose	A, L	A, P, L*	nd	nd	A, L	A, P, S	nd	A, L, P, S	A, L, P	L, P, S	A, L
Major fatty acids (%)	anteiso-C_15:0_ (31.9), C_15:0_ (14.8), C_16:0_ (8.2)	anteiso-C_15:0_ (22.6), C_15:0_ (18.5), iso-C_14:0_ (9.7)*	anteiso-C_15:0_ (34.0), iso-C_14:0_ (24.0), iso-C_16:0_ 3OH (9.4)*	anteiso-C_15:0_ (23.9), C_16:0_ (15.2), iso-C_14:0_ (12.9)*	anteiso-C_15:0_ (31.3), iso-C_17:0_ 3OH (15.0), C_16:0_ 3OH (7.8)*	anteiso-C_15:0_ (35.9), C_18:1_ *ω*9*c* (21.7), C_16:0_ (7.6)	anteiso-C_15:0_ (72.9), C_15:0_ (4.0), C_16:0_ (3.8)	anteiso-C_15:0_ (30.6), C_15:0_ (10.6), iso-C_14:0_ (8.1)	anteiso-C_15:0_ (34.6), C_18:1_ *ω*9*c* (13.1), anteiso-C_17:0_ 3OH (8.1)	iso-C_14:0_ (19.8), anteiso-C_15:0_ (19.6), iso-C_16:0_ 3OH (12.3)*	anteiso-C_15:0_ (18.6), iso-C_14:0_ (15.7), C_16:0_ (15.5)
*nuo* genes	+	+	+	+	+	+	+	+	+	+	+
Cytochrome *bd* oxidase	+	+	+	+	+	+	+	+	+	+	+
*nqr* genes	−	−	−	−	−	−	−	+	−	−	+

*a*: Based on draft genome assembly GCF_000213575.1.

*b*: Based on draft genome assembly GCF_014196915.1.

*c*: Based on draft genome assembly GCF_000213555.1.

*d*: Based on draft genome assembly GCF_900128985.1.

*e*: Based on draft genome assembly GCF_000426485.1.

*Data from Kodama *et al.* [4].

Organic acids of the metabolic end products in PYG broth (1%, w/v, of each) were analysed as described by Pramono *et al.* [6]. The major end products of strains MK137_Hg11^T^ and MK137_Hg34^T^ were lactate (7.6 and 6.4 mM, respectively) and acetate (4.2 and 3.2 mM, respectively). For comparison, *D. macrotermitis* JCM 19375ᵀ produced 6.8 mM lactate, 11.4 mM acetate and 20.3 mM propionate. While *D. macrotermitis* JCM 19375ᵀ produced propionate as a major end product, strains MK137_Hg11ᵀ and MK137_Hg34ᵀ did not.

Fatty acid methyl esters were derived from ~40 mg of wet cells grown on appropriate agar plates at 30 °C for 3 days, through saponification, methylation and extraction, with minor modifications [42] to Miller’s method [43]. Cellular fatty acid profiles were analysed using version 6.2B of the Sherlock Microbial Identification System (midi) and version 3.80 of the BHIBLA database. The major cellular fatty acids (>10%) of strain MK137_Hg11^T^ were anteiso-C_15:0_ (31.9%) and C_15:0_ (14.8%), and those of strain MK137_Hg34^T^ were iso-C_14:0_ (15.7%), anteiso-C_15:0_ (18.6%), C_16:0_ (15.5%), C_18:1_* ω*9*c* (14.1%) and C_18:2_* ω*9,6*c* (10.5%) (Tables 1 and S3).

Although members of *Dysgonomonas* have been described as facultatively anaerobic, the genomic basis underlying their oxygen utilization has not been systematically characterized. Based on Clusters of Orthologous Genes (COG) annotation of *Dysgonomonas* genomes, we found that all examined species possess genes encoding a cytochrome *bd* oxidase, which enables the use of oxygen as a terminal electron acceptor (Table 1). Additionally, all genomes encode at least one set of genes for NADH dehydrogenase (*nuo*). The presence of these core components of an electron transport chain is a common feature within *Dysgonomonas*. Interestingly, we also identified genes for an Na^+^-translocating NADH:quinone reductase (*nqr*) in strain MK137_Hg34^T^ and in *D. alginatilytica*, but not in other species. This suggests some diversity in the strategies for maintaining redox balance and energy conservation within this group.

In contrast to these relatively conserved respiratory components, the distribution of genes for quinone biosynthetic pathways was highly variable, even among species within the same phylogenetic cluster (Table S4). For example, the gene repertoire of strain MK137_Hg11^T^ differed from those of other species in the *D. gadei* group, such as *D. gadei* and *D. hofstadii*. Notably, strain MK137_Hg34ᵀ lacks most of the key genes required for both menaquinone and ubiquinone biosynthesis, implying that it may utilize an alternative or as-yet-uncharacterized pathway for quinone synthesis, which highlights its distinct metabolic traits.

**Fig. 4.**
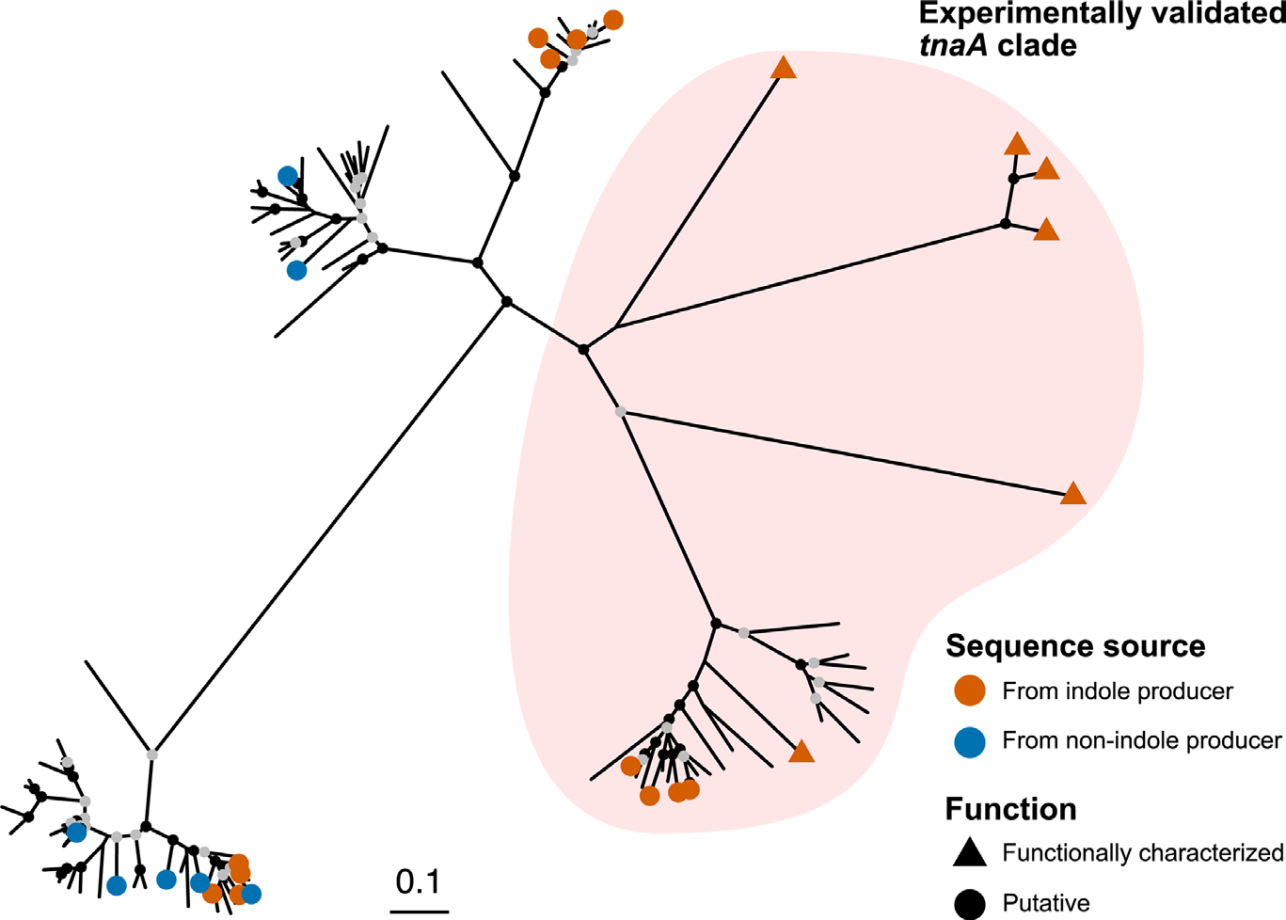
Maximum-likelihood phylogenetic tree of *tnaA* genes identified in isolates and MAGs classified within the family *Dysgonomonadaceae*. The tree was constructed using the LG+I+R4 amino acid substitution model. Experimentally validated sequences with known indole-producing function (UniProt IDs: P0A853, Q6LP64, E9RK37, O30971, P31015, A5EYI0) were included for reference.

**Fig. 5.**
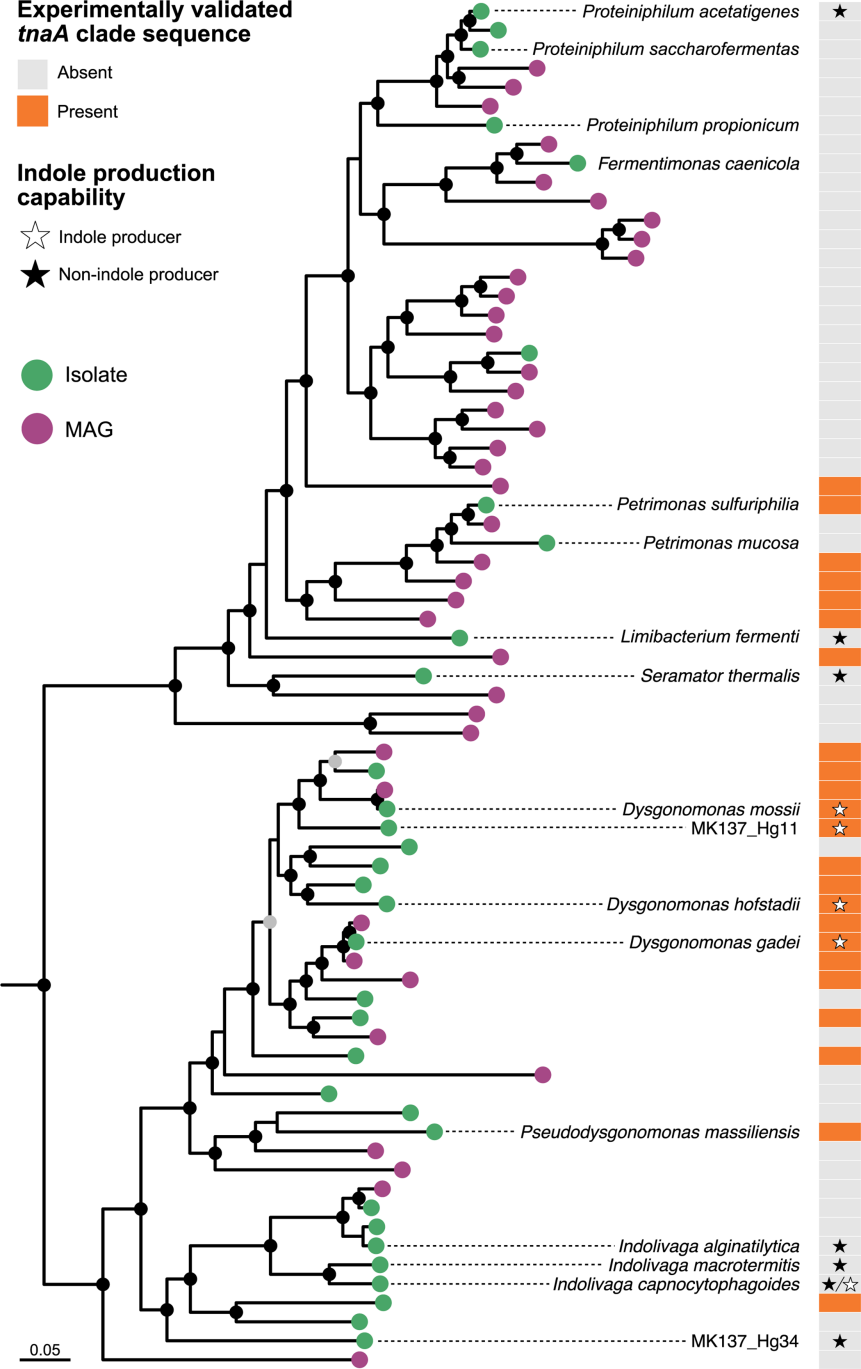
Distribution of sequences belonging to the *tnaA* clade containing experimentally validated indole-producing genes. Maximum-likelihood phylogenomic tree of isolates and MAGs assigned to the family *Dysgonomonadaceae*, constructed from a concatenated alignment of single-copy marker gene amino acid sequences. The tree was constructed using the LG+F+R7 amino acid substitution model and rooted with *Coprobacter fastidiosus* (GCF_000473955.1) and *Coprobacter secundus* subsp. *similis* (GCF_015097275.1) as the outgroup. Nodes with SH-aLRT support values ≥80% and ultrafast bootstrap support values ≥95% are indicated by solid black circles; nodes with either SH-aLRT≥80% or ultrafast bootstrap≥95% are indicated by solid grey circles.

To investigate phenotypic distinctions between the groups that may represent separate genera based on AAI and POCP thresholds, we compared indole production among representative strains (Table 1). Notably, *D. mossii*, *D. hofstadii*, *D. gadei* and strain MK137_Hg11^T^ were positive for indole production, whereas *D. macrotermitis*, *D. capnocytophagoides*, *D. alginatilytica* and strain MK137_Hg34^T^ were negative. The status of *D. capnocytophagoides* is inconsistent in the literature, reported as variable in [1] and negative in BacDive [44]. The variability noted by Hofstad *et al.* [1] stems from early characterizations of the broader CDC group DF-3 [45], a collection of strains whose taxonomic status as a single species was not definitively established at that time. This clade-specific pattern suggests that indole production and the presence of *tnaA*, encoding tryptophanase (COG3033), may serve as an additional taxonomic marker distinguishing these groups. To explore this possibility, we constructed a phylogenetic tree based on *tnaA* sequences (Fig. 4). The phylogenetic tree showed the presence of multiple clades, including one that contained sequences with experimentally validated indole-producing function. *tnaA* sequences from indole-positive strains clustered within this clade, whereas those from indole-negative strains were absent from it. Strains harbouring sequences belonging to the clade containing experimentally validated indole-producing *tnaA* genes were identified on the genome-based phylogenetic tree (Fig. 5). The sequences were from not only *D. mossii*, *D. hofstadii*, *D. gadei* and MK137_Hg11^T^, but also several closely related undescribed strains and MAGs. These findings support the hypothesis that indole production, along with *tnaA* gene phylogeny, may reflect a deeper evolutionary separation and provide additional evidence for genus-level delineation within *Dysgonomonas*.

Furthermore, based on both AAI and POCP thresholds, strain MK137_Hg34^T^ may represent a separate genus not only from *D. mossii*, *D. hofstadii*, *D. gadei* and strain MK137_Hg11^T^, but also from *D. macrotermitis*, *D. capnocytophagoides* and *D. alginatilytica*. These distinctions are further supported by differences in phenotypic characteristics such as the ability to utilize maltose and l-arabinose and the presence or absence of *α*-galactosidase and *β*-glucosidase activities. The proportion of C_18:2_* ω*9,6*c* in the cellular fatty acid profile of MK137_Hg34^T^ exceeds 10%, whereas in other related species it remains below 6%, further highlighting its distinctiveness.

Although not formally described, ‘*D. massiliensis*’ also appears to represent a separate genus based on genome-based criteria. Notably, it differs morphologically from other members of the genus *Dysgonomonas*, exhibiting coccoid cell morphology rather than rod-shaped cells. It also displays distinct phenotypic features, including a smaller cell size and markedly smaller colony size. Unlike other species in the genus, which are all positive for alkaline phosphatase, ‘*D. massiliensis*’ is negative for this enzyme. Furthermore, it lacks *β*-galactosidase activity, in contrast to the *D. gadei* group, to which it forms a sister clade.

To place our findings within a standardized taxonomic context, we analysed the potential generic divisions using the relative evolutionary divergence (RED) metric from the Genome Taxonomy Database (GTDB) framework (Fig. S2). In the GTDB taxonomy (release R220), all members of the *Dysgonomonas* species, strains MK137_Hg11^T^ and MK137_Hg34^T^, are placed within a single genus *Dysgonomonas*, which exhibits a RED value of 0.892. This value is lower than those of neighbouring genera such as *Proteiniphilum* (0.944) and *Petrimonas* (0.971) and also falls below the median RED value for genera across the phylum *Bacteroidota* (0.928), suggesting that the current classification represents a genus with a relatively low evolutionary depth for its rank. In contrast, if the four major phylogenetic and phenotypic groups identified in this study were to be treated as distinct genera, the RED values for these groups would be elevated to a more consistent range of 0.914–0.925. This hypothetical subdivision would bring the evolutionary depth of these taxa much closer to the phylum-wide median. Furthermore, an analysis of the ‘core clades’ – defined here as the smallest clades encompassing all validly published species within the *D. gadei* group and the *D. capnocytophagoides* group – revealed even higher RED values of 0.946 and 0.956, respectively. These values are highly congruent with those of their established neighbours. Taken together, the RED analysis demonstrates that subdividing *Dysgonomonas* species based on its internal phylogenetic structure does not create taxonomically aberrant ranks. Instead, such a revision would serve to harmonize the evolutionary depth of these groups with that of surrounding taxa.

Based on the results presented here, we propose that *D. macrotermitis*, *D. capnocytophagoides* and *D. alginatilytica* should be reclassified into a separate genus, *Indolivaga*. Likewise, *D. massiliensis* should be reclassified in the new genus, *Pseudodysgonomonas*. Strain MK137_Hg11^T^ represents a novel species within the genus *Dysgonomonas*, for which the name *Dysgonomonas reticulitermitis* sp. nov. is proposed. Strain MK137_Hg34^T^ is proposed as the type strain of a new genus closely related to *Dysgonomonas*, for which the name *Viscerimonas tarda* gen. nov., sp. nov. is proposed.

## Description of *Indolivaga* gen. nov.

*Indolivaga* (In.do.li.va’ga. N.L. neut. n. *indolum*, indole; L. masc. adj. *vagus*, wandering, roaming, unfixed; N.L. fem. n. *Indolivaga*, an organism wandering away from indole, implying it does not participate in indole production).

Gram-stain-negative, facultative anaerobic, non-spore-forming, non-motile. Cell morphology is variable, from coccobacilli to rods. *Indolivaga* species generally do not produce indole. The genus is a member of the family *Dysgonomonadaceae*. The type species is *Indolivaga capnocytophagoides*.

## Description of *Indolivaga capnocytophagoides* comb. nov.

*Indolivaga capnocytophagoides* (cap.no.cy.to.pha.go.i’des. Gr. masc. n. *kapnos*, smoke; Gr. neut. n. *kytos*, hollow vessel; Gr. inf. v. *phageîn*, to eat; L. adj. suff. *-oides*, alike; N.L. fem. adj. *capnocytophagoides*, capnocytophaga-like, referring to some properties shared between these organisms).

Basonym: *D. capnocytophagoides* Hofstad *et al.* 2000.

Characteristics of the species are as described [1].

The type strain CCUG 17996^T^ (=CIP 107043^T^=DSM 22835^T^=JCM 16697^T^=LMG 11519^T^) was isolated from human clinical specimens. The G+C content of the genome is 37.5 mol%.

## Description of *Indolivaga macrotermitis* comb. nov.

*Indolivaga macrotermitis* (ma.cro.ter’mi.tis. N.L. gen. n. *macrotermitis*, of the termite *Macrotermes*, where the organism was first isolated).

Basonym: *D. macrotermitis* Yang *et al.* 2014.

Characteristics of the species are as described [7].

The type strain Dys-CH1^T^ (=DSM 27370^T^=JCM 19375^T^) was isolated from the hindgut of a fungus-growing termite *M. barneyi*. The G+C content of the genome is 38.5 mol%.

## Description of *Indolivaga alginatilytica* comb. nov.

*Indolivaga alginatilytica* [al.gi.na.ti.ly’ti.ca. N.L. neut. n. *alginatum*, alginate; N.L. masc. adj. *lyticus*, dissolving (from Gr. masc. adj. *lytikos*, dissolving); N.L. fem. adj. *alginatilytica*, alginate-dissolving].

Basonym: *D. alginatilytica* Kita *et al.* 2015.

Characteristics of the species are as described [2].

The type strain HUA-2^T^ (=DSM 100214^T^=HUT 8134^T^=JCM 38120^T^) was isolated from a sea sand sample. The G+C content of the genome is 37.5 mol%.

## Description of *Pseudodysgonomonas* gen. nov.

*Pseudodysgonomonas* (Pseu.do.dys.go.no.mo’nas. Gr. neut. adj. *pseudes*, false; L. fem. n. *monas*, a unit, monad; N.L. fem. n. *Dysgonomonas*, genus of Gram-negative and fermentative bacteria; N.L. fem. n. *Pseudodysgonomonas*, a false *Dysgonomonas*).

Gram-stain-negative, facultative anaerobic, non-spore-forming, non-motile. Cell morphology is coccus. Alkaline phosphatase negative. The type species is *Pseudodysgonomonas massiliensis*.

## Description of *Pseudodysgonomonas massiliensis* sp. nov.

*Pseudodysgonomonas massiliensis* (mas.si.li.en’sis. L. fem. adj. *massiliensis*, pertaining to Massilia, the ancient name of the city of Marseille, where this bacterium was characterized).

Characteristics of the species are as described [8].

The type strain Marseille-P4356^T^ (=CCUG 71356^T^=JCM 38122^T^) was isolated from a stool sample of a human. The G+C content of the genome is 37.3 mol%. The GenBank/EMBL/DDBJ accession number for the 16S rRNA gene sequence of strain Marseille-P4356^T^ is LT934443. The GenBank/EMBL/DDBJ accession number for the genome sequences of strain Marseille-P4356^T^ is NZ_OEPV00000000.

## Description of *Viscerimonas* gen. nov.

*Viscerimonas* (Vis.ce.ri.mo’nas. L. neut. n. *viscus*, entrails, intestines; L. fem. n. *monas*, a unit, monad, N.L. *Viscerimonas*, a monad inhabiting the intestines).

Gram-stain-negative, facultative anaerobic, non-spore-forming, non-motile. Cell morphology is rod-shaped. The genus is a member of the family *Dysgonomonadaceae*. The type species is *V. tarda*.

## Description of *Viscerimonas tarda* sp. nov.

*Viscerimonas tarda* (tar’da. L. fem. adj. *tarda*, slow or inactive, referring to the slow growth of the type strain).

Cells are 2.0–5.0×0.8 µm in size and occur singly or in short chains of 2–10 cells. Grows at 25–30 °C (optimum, 30 °C) and at pH 6.5–8.0 (optimum, pH 6.5–7.5). Growth doesn’t occur in the presence of Oxgall. Colonies on EG agar after 7 days of incubation at 30 °C under microoxic conditions are 0.1–0.3 mm in diameter, white, circular, convex and opaque. Indole and catalase are not produced. Urease is not produced. Utilizes d-glucose, lactose, l-rhamnose (variable) and d-xylose, but not cellobiose, maltose, l-arabinose, d-mannitol, sucrose, trehalose and CM-cellulose. Positive reactions are obtained using the API ZYM system for acid phosphatase, alkaline phosphatase, esterase, esterase lipase, *β*-galactosidase, *α*-glucosidase and naphthol-AS-BI-phosphohydrolase. Negative reactions for lipase, valine arylamidase, cysteine arylamidase, leucine arylamidase, chymotrypsin, *α*-galactosidase, *α*-fucosidase, *α*-mannosidase, *β*-glucuronidase, *β*-glucosidase, *N*-acetyl-*β*-glucosaminidase and trypsin. Positive reactions are also obtained using Rapid ID32A for alkaline phosphatase, *β*-galactosidase, *α*-glucosidase, *α*-arabinosidase, leucyl glycine arylamidase and alanine arylamidase. Negative reactions for indole production, *α*-galactosidase, *α*-fucosidase, *β*-glucosidase, *N*-acetyl-*β*-glucosaminidase, arginine dihydrolase, 6-phospho-*β*-galactosidase, *β*-glucuronidase, glutamic acid decarboxylase, arginine arylamidase, glutamyl glutamic acid arylamidase, glycine arylamidase, histidine arylamidase, leucine arylamidase, phenylalanine arylamidase, proline arylamidase, pyroglutamic acid arylamidase, serine arylamidase, tyrosine arylamidase, nitrate reduction and urease. Mannose and raffinose are not fermented. The type strain, MK137_Hg34^T^ (=JCM 35195^T^=DSM 118090^T^), was isolated from *R. speratus* gut content. The genome G+C content of the type strain is 43.3 mol%. The GenBank/EMBL/DDBJ accession number for the 16S rRNA gene sequence of strain MK137_Hg34^T^ is LC807697. The GenBank/EMBL/DDBJ accession number for the genome sequences of strain MK137_Hg34^T^ is BAAHWV010000001-BAAHWV010000144.

## Emended description of the genus *Dysgonomons* Hofstad *et al.* 2000

The description is as given by Hofstad *et al.* [1] with the following modification. Indole is generally produced.

## Description of *Dysgonomons reticulitermitis* sp. nov.

*Dysgonomonas reticulitermitis* (re.ti.cu.li.ter’mi.tis. N.L. gen. n. *reticulitermitis*, of *Reticulitermes*, the genus of the termite from which the type strain was isolated).

Cells are 1.5–5.0×0.5 µm in size and occur singly or in short chains of two cells. Grows at 25–30 °C (optimum, 30 °C) and at pH 6.0–8.0 (optimum, pH 7.0–7.5). Growth doesn’t occur in the presence of Oxgall. Colonies on Brucella blood agar after 7 days of incubation at 30 °C under microoxic conditions are 0.2–0.5 mm in diameter, white, circular, convex and opaque. Indole and catalase are produced. Urease is not produced. Utilizes d-glucose, cellobiose, maltose, lactose, sucrose, trehalose and d-xylose, but not l-arabinose, d-mannitol, l-rhamnose and CM-cellulose. Positive reactions are obtained using the API ZYM system for acid phosphatase, alkaline phosphatase, esterase, esterase lipase, *α*-galactosidase, *β*-galactosidase, *α*-glucosidase, naphthol-AS-BI-phosphohydrolase, *N*-acetyl-*β*-glucosaminidase, *α*-fucosidase, *β*-glucosidase, *β*-glucuronidase, leucine arylamidase and trypsin. Negative reactions for lipase, valine arylamidase, cysteine arylamidase, chymotrypsin and *α*-mannosidase. Positive reactions are also obtained using Rapid ID32A for alkaline phosphatase, *α*-galactosidase, *β*-galactosidase, *α*-glucosidase, *β*-glucosidase, *N*-acetyl-*β*-glucosaminidase, *α*-fucosidase, indole production, leucyl glycine arylamidase and alanine arylamidase. Negative reactions for *α*-arabinosidase, arginine dihydrolase, 6-phospho-*β*-galactosidase, *β*-glucuronidase, glutamic acid decarboxylase, arginine arylamidase, glutamyl glutamic acid arylamidase, glycine arylamidase, histidine arylamidase, leucine arylamidase, phenylalanine arylamidase, proline arylamidase, pyroglutamic acid arylamidase, serine arylamidase, tyrosine arylamidase, nitrate reduction and urease. Mannose and raffinose are fermented. The type strain, MK137_Hg11^T^ (=JCM 35194^T^=DSM 118089^T^), was isolated from *R. speratus* gut content. The genome G+C content of the type strain is 39.5 mol%. The GenBank/EMBL/DDBJ accession number for the 16S rRNA gene sequence of strain MK137_Hg11^T^ is LC807696. The GenBank/EMBL/DDBJ accession number for the genome sequences of strain MK137_Hg11^T^ is BAAHWU010000001-BAAHWU010000129.

## Supplementary material

10.1099/ijsem.0.007031 Supplementary Material 1.

## Abbreviations

AAI: average amino acid identity
ANI: average nucleotide identity
CAZymes: carbohydrate-active enzymes
CDSs: coding sequences
COG: Clusters of Orthologous Genes
dDDH: digital DNA–DNA hybridization
GTDB: Genome Taxonomy Database
MAGs: metagenome-assembled genomes
PGAP: NCBI Prokaryotic Genome Annotation Pipeline
POCP: percentage of conserved proteins
PYG: peptone–yeast extract–glucose
RED: relative evolutionary divergence
YCFA: yeast casitone fatty acids
