# Supplementary material for: Reclassification of the genus Dysgonomonas and description of Dysgonomonas reticulitermitis sp. nov. and Viscerimonas tarda gen. nov., sp. nov. from the gut of the subterranean termite Reticulitermes speratus
Source: Int J Syst Evol Microbiol. 2026 Jan 19;76(1):007031. doi: 10.1099/ijsem.0.007031 (PMC12816885; doi:10.1099/ijsem.0.007031)
Supplement: Supplementary Material 1. [file ijsem-76-07031-s001.pdf]

Reclassification of the genus *Dysgonomonas* and two novel species *Dysgonomonas reticulitermitis* sp. nov. and *Viscerimonas tarda* gen. nov., sp. nov., isolated from the gut of the subterranean termite *Reticulitermes speratus*

Kazuki Takahashi[1,2,*,†], Atsushi Hisatomi[2,†], Kosuke Mori[3], Masahiro Yuki[2], Satoko Noda[3,4], Yuichi Hongoh[1,2], Moriya Ohkuma[2] and Mitsuo Sakamoto[2,5,*]

Author affiliations: [1]School of Life Science and Technology, Institute of Science Tokyo, Meguro-ku, Tokyo 152-8550, Japan; [2]Microbe Division/Japan Collection of Microorganisms, RIKEN BioResource Research Center, Tsukuba, Ibaraki 305-0074, Japan; [3]Graduate School of Life and Environmental Sciences, University of Yamanashi, Yamanashi 400-8510, Japan; [4]Graduate School of Science and Engineering, Ibaraki University, Mito, Ibaraki 310-8512, Japan; [5]NODAI Culture Collection Center, Tokyo NODAI Research Institute, Tokyo University of Agriculture, Setagaya-ku, Tokyo 156-8502, Japan.

[†]These authors contributed equally to this work

[*]**Correspondence:** Kazuki Takahahi, kazuki.takahashi.fg@riken.jp; Mitsuo Sakamoto, sakamoto@riken.jp

Supplementary methods

- **Selection of MAGs for AAI and POCP comparisons**

Metagenome-assembled genomes (MAGs) used for AAI and POCP comparisons were retrieved from the GTDB (release R220) [1]. Only genomes with a completeness of ≥90% and contamination of <5%, as assessed by CheckM2, were included in the analysis.

- **Calculation of relative evolutionary divergence (RED) and construction of the RED-normalized tree**

To assess phylogenetic relationships within the family *Dysgonomonadaceae*, the genomes of strains MK137_Hg11^{T} and MK137_Hg34^{T} were integrated into the GTDB bacterial reference tree using the denovo_wf workflow implemented in GTDB-Tk v2.4.0 (based on GTDB release R220) [2]. The resulting phylogenomic tree was further processed with PhyloRank v0.1.12 (https://github.com/donovan-h-parks/PhyloRank) using the outliers command to calculate RED (Relative Evolutionary Divergence) values and to generate a RED-normalized phylogenomic tree. This tree is presented as Fig. S2.

- **Identification of respiratory chain–related genes**

Protein-coding sequences were annotated against the COG database (version 2020) [3] using rpsblast v2.13.0+ [4] with an e-value cutoff of 1e−4. The top hit for each query was retained, and the presence or absence of genes encoding NADH dehydrogenase (*nuo*), cytochrome *bd* oxidase, and Na^{+}-translocating NADH:quinone reductase (*nqr*) was assessed based on COG annotations.

- **Identification of quinone biosynthesis pathway genes**

To detect genes involved in quinone biosynthesis, we used the HMM profiles compiled by Chobert *et al.* [5]. Searches were performed using hmmsearch implemented in HMMER v3.3.2, with an e-value threshold of 1e−4. Genes with hits below this threshold were considered present.

Supplementary figures

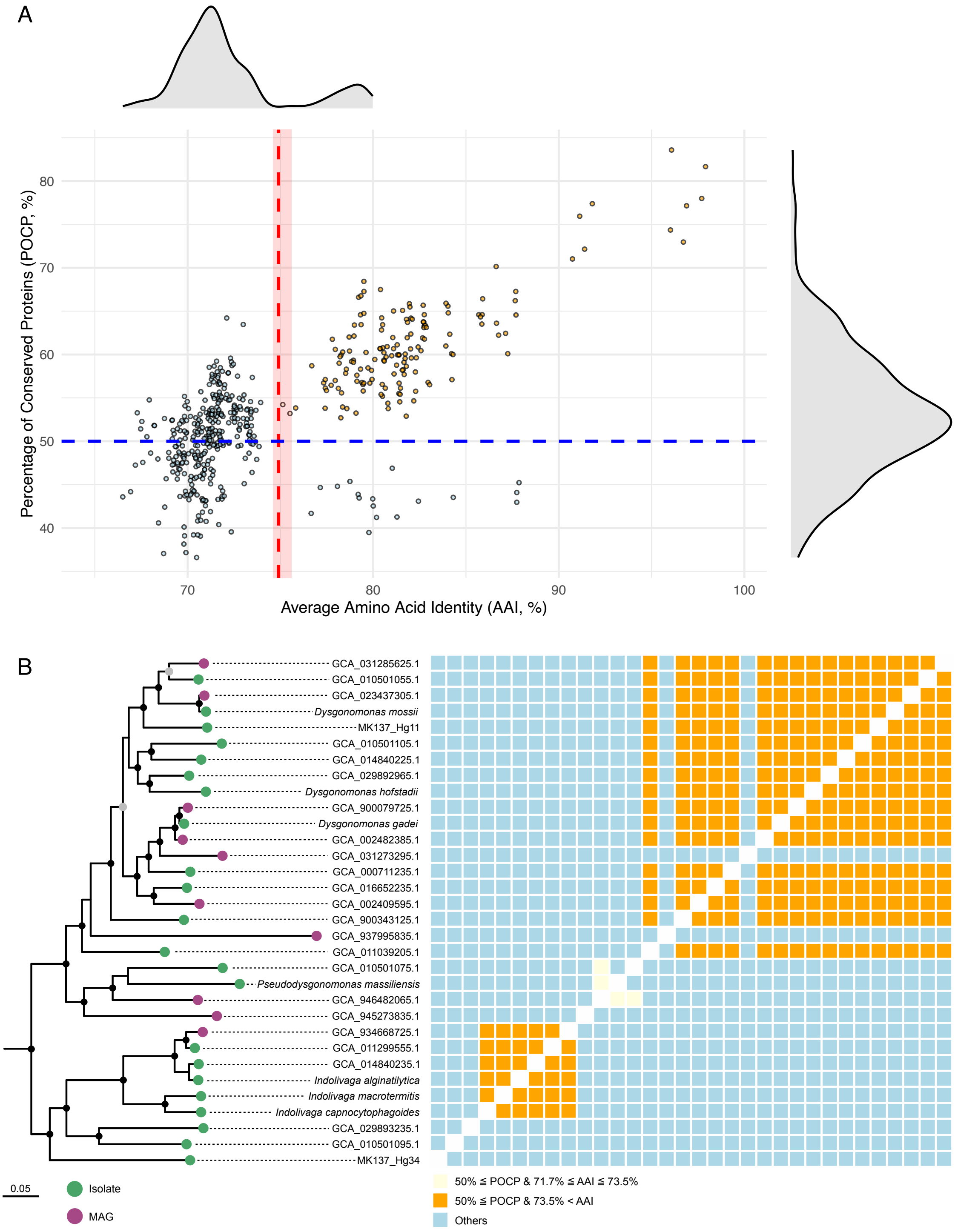

Fig. S1. (**A**) Relationship between AAI of 128 conserved single-copy gene and POCP values based on pairwise whole-genome comparisons. Each point represents a comparison between two genomes. The red dashed line indicates the local minimum of the AAI distribution estimated with kernel density estimation using an adjustment

parameter (*adjust*) of 1. The light red band represents the range of local minima obtained by varying the *adjust* value in the kernel density estimation. (**B**) Maximum-likelihood phylogenomic tree of isolates and metagenome-assembled genomes (MAGs) assigned to the genus *Dysgonomonas*, constructed from a concatenated alignment of single-copy marker gene amino acid sequences. The tree was constructed using the LG+F+R7 amino acid substitution model and rooted with *Coprobacter fastidiosus* (GCF_000473955.1) and *Coprobacter secundus* subsp. *similis* (GCF_015097275.1) as the outgroup. Nodes with SH-aLRT support values ≥80% and ultrafast bootstrap support values ≥95% are indicated by solid black circles; nodes with either SH-aLRT ≥80% or ultrafast bootstrap ≥95% are indicated by solid gray circles. Outer tiles correspond to the AAI–POCP relationship shown in (A), with colors indicating the combined criteria.

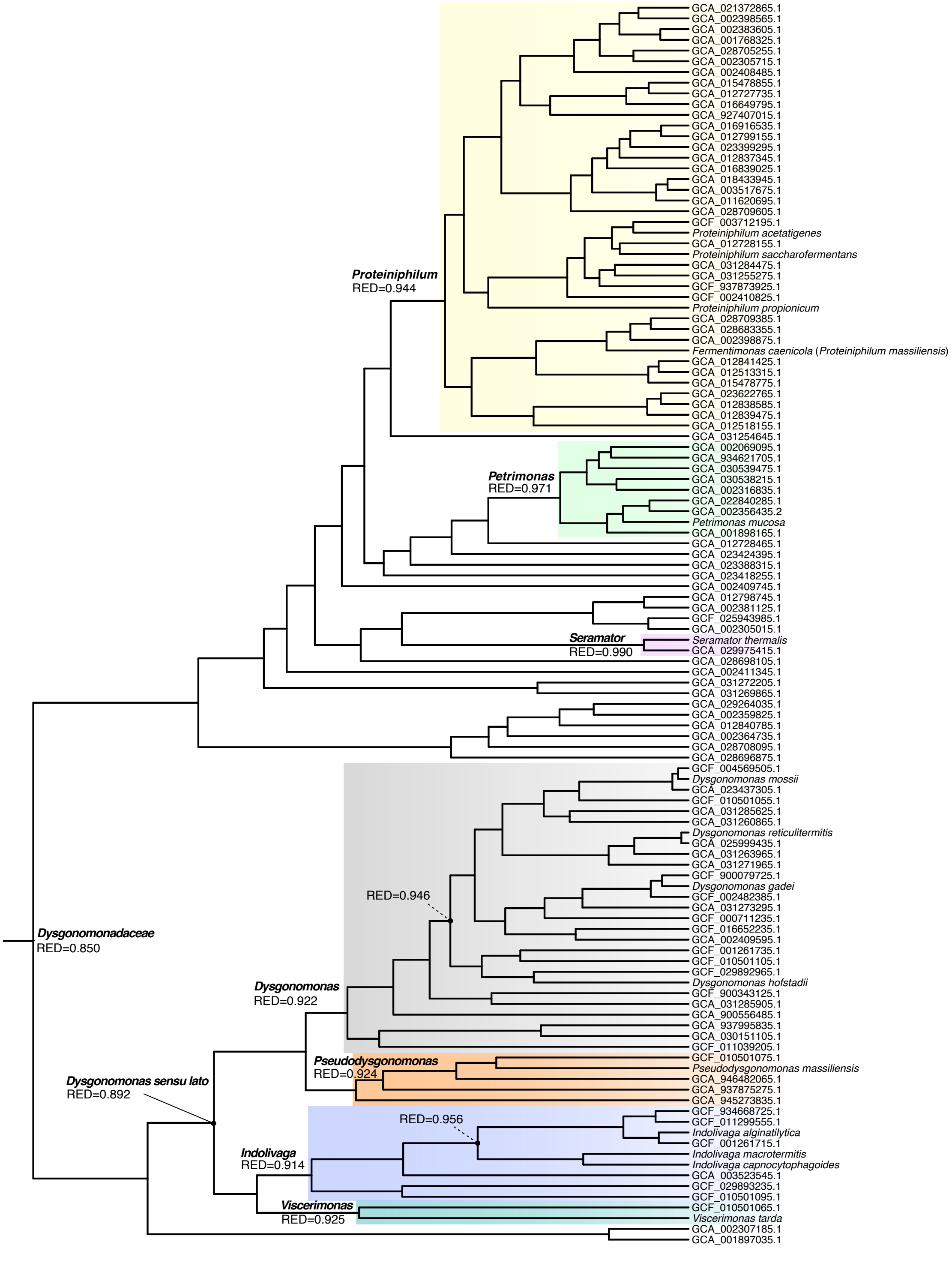

Fig. S2. RED-normalized phylogenomic tree of the family *Dysgonomonadaceae*. RED values are shown for major clades. Labels for the proposed genera (*Dysgonomonas*, *Pseudodysgonomonas*, *Indolivaga*, and *Viscerimonas*) indicate the RED value for the entire monophyletic clade. For *Dysgonomonas* and *Indolivaga*, an additional RED value, indicated by a dotted line, is shown for the core clade (defined as the smallest clades

encompassing all validly published species).

Table S1. The digital DNA-DNA hybridization (top, bold type) and average nucleotide identity (bottom, normal type) values (%) among species of the genus *Dysgonomonas*

	1	2	3	4	5	6	7	8	9
1. MK137_Hg11^T	-	**20.4**	**21.4**	**21.1**	**20.4**	**23.2**	**22.3**	**23.0**	**22.4**
2. *Dysgonomonas mossii* DSM 22836^T	73.9	-	**23.3**	**23.7**	**21.8**	**22.1**	**21.5**	**26.8**	**21.2**
3. *Dysgonomonas hofstadii* JCM 17038^T	73.4	73.8	-	**23.9**	**20.3**	**20.0**	**24.4**	**32.8**	**20.6**
4. *Dysgonomonas gadei* JCM 16698^T	73.7	74.4	74.8	-	**20.0**	**21.7**	**23.0**	**31.2**	**21.5**
5. *Dysgonomonas massiliensis* CCUG 71356^T	69.7	69.7	69.7	69.8	-	**20.0**	**21.3**	**21.9**	**19.8**
6. *Dysgonomonas alginatilytica* DSM 100214^T	70.0	70.4	69.7	70.0	69.2	-	**20.1**	**19.9**	**20.5**
7. *Dysgonomonas macrotermitis* JCM 19375^T	70.0	70.3	70.5	70.2	69.0	72.0	-	**20.6**	**20.5**
8. *Dysgonomonas capnocytophagoides* JCM 16697^T	69.8	70.7	70.9	71.1	69.4	72.1	75.2	-	**20.9**
9. MK137_Hg34^T	69.5	69.4	69.6	69.7	68.2	69.2	69.2	69.3	-

Table S2. Percentage of conserved proteins (top, bold type) and average amino acid identity (bottom, normal type) values (%) among species of the genus *Dysgonomonas*

	1	2	3	4	5	6	7	8	9
1. MK137_Hg11^T	-	**58.2**	**56.7**	**55.5**	**51.1**	**48.0**	**46.3**	**49.5**	**45.8**
2. *Dysgonomonas mossii* DSM 22836^T	77.5	-	**62.8**	**65.9**	**56.5**	**53.8**	**52.6**	**58.5**	**44.4**
3. *Dysgonomonas hofstadii* JCM 17038^T	75.6	76.3	-	**65.7**	**52.1**	**50.8**	**50.7**	**53.6**	**40.8**
4. *Dysgonomonas gadei* JCM 16698^T	76.7	77.3	76.9	-	**53.7**	**54.8**	**53.1**	**58.7**	**43.1**
5. *Dysgonomonas massiliensis* CCUG 71356^T	68.9	68.5	68.4	68.5	-	**47.3**	**48.6**	**51.1**	**42.4**
6. *Dysgonomonas alginatilytica* DSM 100214^T	68.1	68.2	67.7	68.1	66.3	-	**62.1**	**63.4**	**43.3**
7. *Dysgonomonas macrotermitis* JCM 19375^T	68.4	68.6	68.3	68.3	66.3	72.3	-	**70.1**	**43.3**
8. *Dysgonomonas capnocytophagoides* JCM 16697^T	68.3	69.3	68.9	69.7	66.5	72.9	79.3	-	**46.1**
9. MK137_Hg34^T	68.5	68.7	68.3	68.8	66.7	68.8	68.9	68.9	-

Table S3. Cellular fatty acid compositions among species of the genus *Dysgonomonas*

Strains: 1, MK137_Hg11^T; 2, MK137_Hg34^T; 3, *D. macrotermitis* JCM 19375^T

**Fatty acid**	**1**	**2**	**3**
$C_{14:0}$	-	7.8±0.7	-
iso-$C_{14:0}$	6.4±0.6	**15.7±1.7**	-
iso-$C_{15:0}$	1.9±0.2	-	-
$C_{15:0}$ ANTEISO	**31.9±1.7**	**18.6±1.6**	**34.6±6.1**
$C_{15:0}$ 3OH	2.7±0.8	-	-
$C_{15:0}$	**14.8±1.8**	3.2±0.3	3.8±1.2
$C_{16:0}$	8.2±1.3	**15.5±2.5**	7.9±1.7
$C_{16:0}$ 3OH	2.7±0.5	4.3±0.9	-
$C_{17:0}$	1.8±0.3	-	-
$C_{17:0}$ ANTE 3OH	1.7±0.4	-	8.1±2.0
$C_{18:0}$	-	3.0±0.8	2.6±1.2
$C_{18:1}\omega 9c$	5.1±1.2	**14.1±1.5**	**13.1±4.2**
$C_{18:2}$ $\omega 9,6c$	5.4±1.1	**10.5±1.3**	3.3±0.8
Summed Feature 1 ($C_{13:1}\omega 1c$ and/or $C_{14:0}$ ALDE)	1.6±0.3	-	-
Summed Feature 3 (iso-$C_{15:0}$ aldehyde and/or UN 13.570)	1.8±1.0	-	4.1±1.4
Summed Feature 9 (iso-$C_{17:1}\omega 9c$ and/or 10-methyl $C_{16:0}$)	5.0±1.3	-	-
Summed Feature 10 ($C_{18:1}$c11/t9/t6 and/or UN 17.834)	-	-	4.7±1.9
Summed Feature 11 ($C_{17:0}$ iso 3-OH and/or $C_{18:2}$ DMA)	2.5±0.8	-	7.2±2.2

Table S4. Distribution of genes for quinone biosynthetic pathways. Presence of genes is indicated by a black circle (●); absence is indicated by a blank cell.

	Ubiquinone biosynthetic pathway												Classical menaquinone biosynthetic pathway								Futalosine pathway			
	UbiC	UbiA	UbiX	UbiD	UbiM	UbiL	UbiI	UbiG	UbiH	UbiE	Coq7	UbiF	MenF	MenD	MenH	MenC	MenE	MenB	MenA	MenG	MqnA	MqnB	MqnC	MqnD
1. MK137_Hg11^{T}										●					●		●		●				●	
2. *Dysgonomonas mossii* DSM 22836^{T}						●	●		●	●							●		●					
3. *Dysgonomonas hofstadii* JCM 17038^{T}							●	●	●	●			●	●	●	●	●	●	●				●	
4. *Dysgonomonas gadei* JCM 16698^{T}									●	●			●	●	●	●	●	●	●					
5. *Dysgonomonas massiliensis* CCUG 71356^{T}										●			●	●		●	●	●	●					
6. *Dysgonomonas alginatilytica* DSM 100214^{T}						●			●	●			●	●	●	●	●	●	●					
7. *Dysgonomonas macrotermitis* JCM 19375^{T}									●	●			●	●		●	●	●	●					
8. *Dysgonomonas capnocytophagoides* JCM 16697^{T}								●	●	●			●	●		●	●	●	●					
9. MK137_Hg34^{T}																	●		●					

References to Supplementary Materials

1. **Parks DH, Chuvochina M, Waite DW, Rinke C, Skarshewski A, *et al.*** A standardized bacterial taxonomy based on genome phylogeny substantially revises the tree of life. *Nat Biotechnol* 2018;36:996–1004.

2. **Chaumeil PA, Mussig AJ, Hugenholtz P, Parks DH**. GTDB-Tk: a toolkit to classify genomes with the Genome Taxonomy Database. *Bioinformatics* 2019;36:1925–1927.

3. **Galperin MY, Wolf YI, Makarova KS, Vera Alvarez R, Landsman D, *et al.*** COG database update: focus on microbial diversity, model organisms, and widespread pathogens. *Nucleic Acids Res* 2021;49:D274–D281.

4. **Camacho C, Coulouris G, Avagyan V, Ma N, Papadopoulos J, *et al.*** BLAST+: architecture and applications. *BMC Bioinformatics* 2009;10:421.

5. **Chobert S-C, Roger-Margueritat M, Flandrin L, Berraies S, Lefèvre CT, *et al.*** Dynamic quinone repertoire accompanied the diversification of energy metabolism in *Pseudomonadota*. *ISME J* 2025;19:wrae253.
